# Beef Production: Koneswaran and Nierenberg Respond

**DOI:** 10.1289/ehp.11716R

**Published:** 2008-09

**Authors:** Gowri Koneswaran, Danielle Nierenberg

**Affiliations:** The Humane Society of the United States, Washington, D.C., E-mail: gkoneswaran@humanesociety.org

Avery and Avery, who find comparing conventional Japanese and organic Swedish beef production misleading, propose relying on “comprehensive life cycle analyses” (LCAs) to quantify emissions from conventional U.S. beef production. However, neither study they cite (Johnson et al. 2003; Subak 1999) appears to be a comprehensive LCA, and it is unclear whether these studies considered emissions created by facets of beef production such as feed transport or pesticide manufacturing, as did Ogino et al. (2007). Additionally, contrary to Avery and Avery’s conclusion, Subak (1999) stated that

These results indicate that the intensification of beef production systems may be counterproductive because net emissions of carbon dioxide as well as nitrogen and other pollutants would increase.

For a more comprehensive analysis, additional production aspects must be considered. Ogino et al. (2007), for example, included the transportation of feed (> 18,000 km, not miles, as stated by Avery and Avery in their letter), which accounted for 8.3% of emissions.

A better comparison of conventional versus organic beef production may be an LCA of greenhouse gas (GHG) emissions from three Irish systems reported by Casey and Holden (2006). Conventional production generated the most GHGs, followed by agri-environmental, with the organic system producing the least GHGs.

In contrast to conventional production, organic farming can reduce nitrous oxide emissions by avoiding excessive amounts of manure, as stocking densities are limited to land available for manure application. Organic agriculture typically also uses less fossil-fuel energy, in part because thousands of feed transport miles may be reduced (Kotschi and Müller-Sämann 2004).

Pasture-based systems require less operational fuel and feed than do conventional systems, and they adeptly sequester GHGs in the soil, tying up 14–21 million metric tons of carbon dioxide and 5.2–7.8 million metric tons of N_2_O in pasture soil organic matter (Boody et al. 2005; Rayburn 1993).

Dourmad et al. (2008) concurred with our conclusion (Koneswaran and Nierenberg 2008) that more research is needed and noted that existing LCAs often omit details such as land-use change information. Many LCAs—and other attempts to quantify GHGs from various systems (Avery and Avery 2007)—also lack data on pesticide use and animal transport from farms or feedlots to slaughter.

In our article (Koneswaran and Nierenberg 2008), we not only argued for refinement of agricultural practices but also for a concurrent reduction in animal product consumption in high-income nations, especially because the U.N. Food and Agriculture Organization has concluded that animal agriculture accounts for more GHGs than transport (Steinfeld et al. 2006). In addition to lowering GHG emissions, reducing animal product consumption could also decrease the incidence of cardiovascular disease, certain cancers, and obesity (McMichael et al. 2007). Given the developing global food crisis, it is important to note, as did Baroni et al. (2007) in the *European Journal of Clinical Nutrition*, that plant-based diets “could play an important role in preserving environmental resources and in reducing hunger and malnutrition in poorer nations.”

Although Avery remains skeptical over the role of anthropogenic GHG emissions in global warming (2008), the Intergovernmental Panel on Climate Change (IPCC 2007) concluded that

Most of the observed increase in global average temperatures since the mid-20th century is very likely due to the observed increase in anthropogenic GHG concentrations.

The link between GHG mitigation and organic or extensive animal agriculture systems is well established, as are the other environmental and public health benefits of less-intensive production systems. Understanding the efficacy of less technology-dependent mitigation strategies is critical as the effects of global warming become more evident.
